# Effectiveness and cost-effectiveness of a very brief physical activity intervention delivered in NHS Health Checks (VBI Trial): study protocol for a randomised controlled trial

**DOI:** 10.1186/s13063-016-1413-2

**Published:** 2016-06-27

**Authors:** Joanna Mitchell, Wendy Hardeman, Sally Pears, Joana C. Vasconcelos, A. Toby Prevost, Ed Wilson, Stephen Sutton, On behalf of the VBI Research Team

**Affiliations:** Behavioural Science Group, Department of Public Health and Primary Care, Institute of Public Health, Forvie Site, University of Cambridge School of Clinical Medicine, Box 113, Cambridge Biomedical Campus, Cambridge, CB2 0SR UK; School of Health Sciences, University of East Anglia, Norwich Research Park, Norwich, NR4 7TJ UK; Imperial Clinical Trials Unit, Imperial College London, Stadium House, 68 Wood Lane, London, W12 7RH UK; Department of Public Health and Primary Care, Institute of Public Health, Forvie Site, University of Cambridge School of Clinical Medicine, Box 113, Cambridge Biomedical Campus, Cambridge, CB2 0SR UK

**Keywords:** Accelerometry, Cost-effectiveness analysis, Pedometer, Physical activity, Primary health care, Randomised controlled trial, Very brief intervention

## Abstract

**Background:**

Physical activity interventions that are targeted at individuals can be effective in encouraging people to be more physically active. However, most such interventions are too long or complex and not scalable to the general population. This trial will test the effectiveness and cost-effectiveness of a very brief physical activity intervention when delivered as part of preventative health checks in primary care (National Health Service (NHS) Health Check).

**Methods/design:**

The Very Brief Intervention (VBI) Trial is a two parallel-group, randomised, controlled trial with 1:1 individual allocation and follow-up at 3 months. A total of 1,140 participants will be recruited from 23 primary care practices in the east of England. Participants eligible for an NHS Health Check and who are considered suitable to take part by their doctor and able to provide written informed consent are eligible for the trial. Participants are randomly assigned at the beginning of the NHS Health Check to either 1) the control arm, in which they receive only the NHS Health Check, or 2) the intervention arm, in which they receive the NHS Health Check plus ‘Step It Up’ (a very brief intervention that can be delivered in 5 minutes by nurses and/or healthcare assistants at the end of the Health Check). ‘Step It Up’ includes (1) a face-to-face discussion, including feedback on current activity level, recommendations for physical activity, and information on how to use a pedometer, set step goals, and monitor progress; (2) written material supporting the discussion and tips and links to further resources to help increase physical activity; and (3) a pedometer to wear and a step chart for monitoring progress.

The primary outcome is accelerometer counts per minute at 3-month follow-up. Secondary outcomes include the time spent in the different levels of physical activity, self-reported physical activity and economic measures.

Trial recruitment is underway.

**Discussion:**

The VBI trial will provide evidence on the effectiveness and cost-effectiveness of the Step It Up intervention delivered during NHS Health Checks and will inform policy decisions about introducing very brief interventions into routine primary care practice.

**Trial registration:**

ISRCTN Registry, ISRCTN72691150. Registered on 17 July 2014.

**Electronic supplementary material:**

The online version of this article (doi:10.1186/s13063-016-1413-2) contains supplementary material, which is available to authorized users.

## Background

Vascular disease, which includes coronary heart disease, stroke, type 2 diabetes and kidney disease, affects more than four million people and causes one out of three deaths and one out of five hospital admissions in England [1]. Physical inactivity is an important risk factor, not only for these diseases but for some cancers too, and is the fourth leading cause of death worldwide [2, 3]. Physical inactivity has been estimated to cause 9 % of premature mortality worldwide. However, if physical activity (PA) interventions increased PA by just 10 %, more than 533,000 deaths could be averted each year [3]. The financial burden from physical inactivity to the National Health Service (NHS) has been estimated at £1.06 billion annually [1], although these costs increase significantly when the wider economic costs are considered [4].The chief medical officer (CMO) for England [1] recommends that adults should take 30 minutes of moderate intensity PA, e.g. brisk walking, on at least 5 days per week. However, the majority of adults in the UK do not meet this recommendation [5], and globally, physical inactivity is on the rise [3].

The National Institute for Health and Care Excellence (NICE) public health guidance endorses brief PA interventions in primary care [4]. When brief advice is compared with usual care, the incremental cost-effectiveness ratio (ICER) of moving one person from an inactive to active state has been estimated at £1730 [4], although this figure is dependent on the setting, content of the PA advice given and the healthcare practitioner delivering the PA intervention [6]. Therefore, brief advice on PA promotion can be cost-effective when the longer-term health benefits and costs are considered [4, 6–8]. In recent years, emphasis has been placed on promoting PA interventions along the continuum of individual-level and population-based interventions. However, time and cost are still significant constraints on their implementation [4].

Very brief interventions (VBIs), defined as interventions delivered in a single session of no more than 5 minutes, could be delivered in primary care consultations such as NHS health checks and annual disease reviews [9, 10]. Very few VBIs that promote PA have been reported in the literature, but where they have, their content is poorly characterised [9–11]. Few studies provide a definition of ‘brief’ or report the duration of the intervention [8, 11]. Furthermore, many ‘brief’ interventions are too long or complex to be cost-effective [6–8, 11–13].

The heterogeneity in methodology and economic modelling further limits the generalisability of results and estimates of cost-effectiveness of PA interventions; usual care is not always the comparator, and relevant costs are often excluded (e.g. out of pocket expenses may be significant in the adoption and maintenance of PA [6]). Measures of effectiveness also vary considerably between studies and are often based on self-reports [5, 12, 13]. Self-report PA questionnaires provide useful information about the types of activities that participants engage in and can be regarded as complementary to objective measures such as those derived from accelerometry. However, self-reports are subject to recall and social desirability biases, which may lead to over-reporting of PA levels [12]. For example, in the 2008 report of the Health Survey for England, the proportions of men and women, respectively, who met the current recommendations for PA were 39 % and 29 % when measured by self-report and 6 % and 4 % when measured by accelerometer [14].

More intensive interventions may be effective at increasing PA, but the most cost-effective interventions at the population level are still likely to be those that require minimal contact [13]. Therefore, an urgent need exists for more clearly specified PA interventions that are scalable, effective and cost-effective.

Our 5-year research programme aims to develop and evaluate very brief interventions that are feasible for primary care consultations such as NHS Health Checks, which are available to adults in England between 40 and 74 years of age. The Health Check includes measurement of cholesterol, blood pressure and BMI to assess a person’s risk of developing vascular disease over 10 years. Therefore, as their primary purpose is to assess and manage risk, Health Checks provide an ideal platform from which to deliver a very brief PA intervention to a large population of adults.

We have developed, piloted and evaluated a number of VBIs [10], and a randomised controlled trial (ISRCTN02863077) of three promising VBIs has been undertaken to determine which intervention to take forward for further testing (Pears S, Bijker M, Morton K, Vasconcelos J, Parker RA, Westgate K, et al.: A randomised controlled trial of three very brief interventions for physical activity in primary care, submitted). Consistent with systematic reviews and meta-analyses of pedometer-based interventions [15], the pedometer-plus VBI was considered to be the most practical and feasible and had the greatest potential to promote change in PA.

The pedometer-plus intervention evaluated in the previous trial involved a face-to-face discussion on the PA recommendations and how to use a pedometer and to set goals and monitor progress. The written material supported the discussion, and it also provided tips to help increase PA. However, although participants and practitioners felt that the length of the intervention was about right, participants thought that the intervention was too generic and would like to have seen a more tailored approach. We therefore refined the intervention, without increasing its duration, to make it more tailored to individuals by emphasising in practitioner training the importance of (1) providing feedback on participants’ current PA and (2) emphasising that any increase in steps per day would be beneficial. The booklet used in the previous trial was refined to include (1) written feedback on the participant’s current PA level; (2) emphasis that any increase in steps per day is beneficial; and (3) information on where to find out about local walking groups and activities and how to download a pedometer application for smartphones.

The pedometer-plus VBI (Step It Up), delivered at the end of the NHS Health Check, is to be tested in this randomised controlled trial against the NHS Health Check alone.

### Aims

The aims of this trial are as follows:

1. To estimate the effectiveness of a very brief pedometer-based intervention (Step it Up) in increasing objectively-measured PA in adults 40–74 years of age attending NHS Health Checks in primary care compared with the Health Check alone
2. To estimate the cost-effectiveness of this intervention compared with the Health Check alone from the perspectives of the NHS and society
3. To assess the mechanisms underlying any intervention effects and the fidelity of delivery

## Methods/design

### Design of the study

**Fig. 1:**
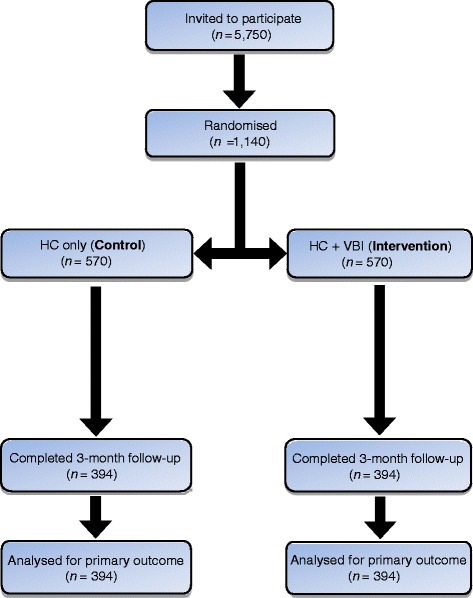
Flow diagram for the VBI Trial. With target or estimated numbers. HC, Health Check; VBI, Very Brief Intervention

The VBI Trial is a two parallel-group, randomised, controlled trial with a 1:1 individual allocation, comparing the Step It Up intervention delivered in an NHS Health Check (intervention arm) with the NHS Health Check alone (usual care control arm). Follow-up is at 3 months. The design of the study and the flow of participants are shown in Fig. 1.

### Eligibility criteria

Inclusion criteria for the trial map onto those for the NHS Health Check. Patients aged between 40 and 74 years, who have not been diagnosed with a vascular disease and are not currently being treated for relevant risk factors (e.g. raised blood pressure) are eligible for an NHS Health Check [2] and, therefore, eligible for inclusion in the trial. However, patients unable to provide written informed consent (e.g. have an insufficient grasp of the English language to understand study procedures) will be excluded, as will those patients whose GP considers them to be unsuitable for inclusion (e.g. because of severe mental impairment or terminal illness).

### Practice recruitment and training

Twenty-three primary care practices in urban and rural areas across the East of England are recruiting 1,140 participants (12 GP practices in Cambridgeshire, eight in Hertfordshire and Bedfordshire and three in Norfolk). Initial contact with the practices is through the regional research network, CRN Eastern. Healthcare practitioners (nurses and/or healthcare assistants) already trained to deliver NHS Health Checks attend a single 3-hour study training session.

Health practitioners receive a training manual as part of the training process, and this, along with the face-to-face session, covers the following: (1) information about the study aims; (2) information about the importance of promoting PA among adults attending NHS Health Checks; (3) study documentation, written materials for the participant, and information on obtaining written informed consent and completing the case report form (CRF) following Good Clinical Practice guidelines; (4) how to access and use the web-based randomisation tool to randomly allocate participants to intervention arm ; (5) a detailed procedure on the Step It Up intervention that describes how each component of the intervention should be delivered, a shortened version of the procedure that practitioners can use as a prompt during the NHS Health Check, and a script which gives an example of intervention delivery; (6) a demonstration and practise of patient-centred communication skills to facilitate behaviour change; (7) role play by the research team using the script in the training manual to demonstrate the NHS Health Check when all study and intervention procedures are incorporated; and (8) practise of the NHS Health Check including all procedures by the healthcare practitioners to consolidate their learning and for the research team to provide feedback on their performance.

To promote fidelity of delivery of the Step It Up intervention, practitioners are trained to use the brief procedure, the CRF and the Step It Up intervention booklet to guide them through the NHS Health Check and intervention delivery.

### Participant recruitment

Recruitment is through the NHS HealthCheck programme [16]. In order to reduce selection bias, a subsample of eligible patients is randomly selected from each participating GP practice. Evidence from our previous trial suggests a likely 20 % response; therefore, 250 invitations are expected to yield 50 participants. Where the response is lower than expected, further subsamples will be generated.

#### Participant invitation

In addition to the invitation for the NHS Health Check, an invitation pack that includes the trial information sheet, an invitation letter and a sample consent form is sent through the mail to patients. Patients are encouraged to make an appointment for the NHS Health Check with their practice, mentioning at the time their interest in taking part in the trial. If, after 2 weeks, no response has been received from the patient, a reminder letter is sent.

Mailing information packs through the post with the NHS Health Check invitation is the preferred method of recruitment. However, based on procedures used in our previous trial, GP practices will have the option to use an alternative method of recruitment as follows: either (1) mail the study information once a patient has made an appointment for the NHS Health Check or (2) provided that patients are given sufficient time to read the information, hand it to patients in the waiting room as they arrive for their NHS Health Checks.

### Study interventions

#### Control arm

Participants in the control arm receive only the usual NHS Health Check. The NHS Health Check is a series of tests and questions designed to assess the individual's risk of developing heart disease, stroke, diabetes and kidney disease. 

#### Intervention arm

Participants in the intervention arm receive the Step It Up intervention in addition to the NHS Health Check. Step It Up is a single-contact intervention that is delivered by nurses and/or healthcare assistants in 5 minutes at the end of the NHS Health Check. It consists of the following three components:

1. Face-to-face discussion: The practitioner (a) gives the patient feedback on their current activity; (b) gives information about the current PA recommendations, as advised by the CMO (30 minutes of moderate-intensity activity on 5 or more days a week or 10,000 steps per day); (c) shows the patient how to wear and use the pedometer and encourages them to use it to monitor the number of steps walked each day; (d) shows the patient the Step Chart and encourages them to use it to set a step goal and record daily steps to monitor whether they reached that goal; and (e) explains that the Pedometer Booklet gives tips for how to increase daily steps by making small changes.
2. Pedometer and Step Chart: A Yamax Digiwalker SW200 and a printed Step Chart are given to the patient to use as self-monitoring tools.
3. Step It Up Booklet: The booklet (a) states the UK government PA recommendations; (b) includes instructions on how to use the pedometer; (c) mentions the health benefits of being more active; (d) provides a graph to show that small changes in PA can lead to significant health benefits; (e) provides tips for achieving more steps; and (f) provides links to other PA resources.

The key behaviour change techniques (BCT) in the Step It Up intervention, identified from the Behaviour Change Technique Taxonomy version 1 [17], include goal setting, action planning, feedback and self-monitoring of behaviour. Table 1 provides a full list and description of the BCTs identified.

**Table 1:** Content and behaviour change techniques (BCT) of the Step It Up very brief intervention

Content of the face-to-face discussion	BCTs^a^ included in the face-to-face discussion
Practitioner	Target behaviour: physical activity
• Gives verbal feedback on current PA and informs the participant of whether they are meeting the PA recommendations	1.1 Goal setting (behaviour)
• Asks the participant if they are aware of the Chief Medical Officer’s PA recommendations and informs them that the recommendations are for a minimum of 30 minutes of moderate-intensity activity on 5 or more days of the week, and emphasises that moderate PA is any activity that raises heart rate, breathing or sweating and includes the activities of daily living	1.4 Action Planning
• Explains the 10,000 steps per day recommendation	2.2 Feedback on behaviour
• Shows the participant how to use the pedometer, and encourages them to use it to monitor daily steps	2.3 Self-monitoring of behaviour
• Shows the participant the Step Chart and encourages them to use it to set a daily step goal (starting with a smaller goal) and record daily steps	8.7 Graded tasks
• Explains that the Step It Up Booklet contains information about the health, social, environmental and emotional benefits of PA; tips for how to increase daily steps by making small changes; and information about other helpful resources.	12.5 Adding objects to the environment
	Target behaviour: Self-monitoring
	4.1 Instruction on how to perform the behaviour
Content of participant materials	BCTs^a^ included in the participant materials
Step It Up Booklet containing	Target behaviour: physical activity
• Written feedback on current PA.	1.1 Goal setting (behaviour)
• Information on PA recommendations (30 minutes of moderate-intensity activity on 5 or more days a week; 10,000 steps per day).	1.4 Action Planning
• Instructions on how to use the pedometer and how to self-monitor daily steps.	2.2 Feedback on behaviour
• Information about the health, social, environmental and emotional benefits of PA.	2.3 Self-monitoring of behaviour
• Advice about setting a smaller step goal at first and gradually increasing the goal over time.	5.1 Information about health consequences
• Tips for how to increase daily steps by making small changes.	5.3 Information about social and environmental consequences
• Information about other helpful online resources (e.g. where to download a pedometer app, a website to find a local walking group).	5.6 Information about emotional consequences
Step Chart	8.7 Graded tasks
• Chart for setting step goals and monitoring daily steps.	Target behaviour: Self-monitoring
Pedometer	
• A Yamax Digiwalker SW200.	4.1 Instruction on how to perform the behaviour

^**a**^Numbering refers to the Behaviour Change Technique Taxonomy v1 [17]

*PA* physical activity

### Procedure

#### Randomisation

Randomisation is stratified by primary care practice using a web-based tool (www.sealedenvelope.com) designed specifically for the trial. The random allocation ratio is 1:1 with randomly permuted blocks of sizes 2, 4 and 6 to ensure even randomisation and low predictability of assignment within each stratum.

The nurse or healthcare assistant uses the web-based program to randomly allocate the patient to either the control arm (the usual NHS Health Check) or the intervention arm (the usual NHS Health Check with the Step It Up intervention). Up until this point, the practitioner and participant are blind to allocation. However, once allocation is known, practitioners are encouraged to convey the information positively by describing allocation in terms of ‘you have been selected to receive the health check today’ (control arm) or ‘you have been selected to receive the Step It Up intervention, which we will do at the end of the health check’ (intervention arm).

#### The NHS Health Check

Healthcare assistants and nurses conducting the NHS Health Checks obtain informed consent from participants following Good Clinical Practice guidelines [18]. Once consent has been given, participants are asked to complete a short questionnaire. The information provided in this questionnaire will be used to characterise the sample and check baseline comparability between arms. The healthcare practitioner accesses the web-based randomisation tool at this time to determine to which intervention arm the participant has been assigned. The rest of the consultation proceeds following the usual NHS Health Check procedures, and the Step It Up intervention is delivered at the end to participants in the intervention arm.

Healthcare practitioners also complete a CRF, which is a written record of the consultation. It is intended as a guide and provides prompts for each stage of the consultation. One additional piece of information is collected: the baseline PA level. This measure is derived during the NHS Health Check using the GP PA Questionnaire (GPPAQ) [19] and is used as the basis for individual feedback at the start of the Step it Up intervention as well as providing a baseline measure of PA. The questionnaire asks about the patient’s PA at work and leisure time in the past week.

#### Audio-recording of consultations

A random sample of five NHS Health Checks (10 %) will be selected from each practice for audio-recording and will include both intervention and control consultations. This should provide a total of 115 audio-recordings. Participants will be asked to confirm their consent verbally at the start of each audio-recording. Neither the practitioner nor the study team will know in advance which consultations are to be audio-recorded (this is determined at the same time as allocation to intervention arm).

#### Follow-up

All participants are followed up 3 months after the Health Check. Participants are asked to wear an accelerometer (Actigraph GT3X+ or Actigraph w-GT3X-BT) 1 around their waist for a period of 7 consecutive days, putting it on in the morning and taking it off before going to bed. Participants are encouraged to go about their activities as normal during this time but are asked to log the times of wear during the day (i.e. the time the monitor was put on and taken off) on the log sheet provided. At the end of the 7 days, participants return the accelerometer in the reply-paid envelope provided along with the log sheet and a completed questionnaire. Based on daily variability findings from the previous trial (Pears S, Bijker M, Morton K, Vasconcelos J, Parker RA, Westgate K, et al.: A randomised controlled trial of three very brief interventions for physical activity in primary care, submitted), a minimum of 3 days of activity data each with a minimum of 10 hours of wear-time data are required to count as a valid recording regardless of how many of the provided days are weekend days or weekdays. Participants are asked to re-wear the accelerometer if the returned data fail to reach this minimum. Non-wear time is determined by 90 minutes or more of zero counts.

Participants will also be asked to complete a questionnaire once they have finished wearing the accelerometer. The questionnaire consists of three parts:

- *Recent physical activity questionnaire (RPAQ)*. Self-reported PA over the previous 4 weeks is measured across four domains (home, work, travel and recreation). Section A on home activities asks questions on the frequency of television viewing, computer use and stair climbing. Section B asks questions about the type of work and the time spent there, as well as assessing the mode of travel to and from place of work. The final section asks about the frequency and duration of frequently performed recreational activities. When tested against two gold standard methods of obtaining PA energy expenditure (PAEE) (doubly labelled water and combined heartrate and movement sensing), the RPAQ has been shown to be a valid tool for ranking individuals according to PAEE [20].
- *NHS use, workplace productivity and expenditure on PA questionnaire*. A bespoke questionnaire on primary and secondary care contacts and expenditure on health, sports clubs or other physical activities was developed and piloted prior to the trial. Work place productivity is based on an adapted version of the validated Work Productivity and Activity Impairment (WPAI) Questionnaire [21].
- *Process evaluation*. (1) For mechanisms of impact, a series of questions is used to assess whether the intervention and materials were received by participants during the NHS Health Check and subsequently used, and (2) for contamination, participants are asked if they know anyone else who has taken part in the study, and if yes, whether they were in the intervention or control group.

#### Maximising retention

To maximise the response rate, everyone who returns the accelerometer with valid data and a completed questionnaire will be entered in a prize draw to receive one of twenty £20 gift vouchers. Participants are also given a study ID card and pen at baseline to remind them of their involvement in the study. To ensure that timing is convenient to receive the accelerometer, participants are contacted (by text, telephone or email) 1 week before the accelerometer and questionnaire are mailed.

#### Data management/quality assurance

The randomisation sequence for the web-based randomisation tool has been generated independently of the study team. Electronic randomisation records will be checked against the CRF for fidelity.

The administrative database is managed in-house. The database has been designed to allow easy entry of CRF data and the short baseline questionnaire. Random checks are performed on the entered data against paper records, and all errors are logged and corrected.

Double data entry of the follow-up questionnaire will be done by an experienced independent agency. The resulting spreadsheets will be interrogated for invalid values and corrected. In addition, random checks will be applied as above.

The objective PA data is checked weekly for completeness, and participants are asked to wear the Actigraph monitor for extra days if the minimum wear time of 10 hours a day over a 3-day period has not been achieved.

The audio-recordings will be submitted to an independent transcription agency and stored on a secure drive on a University server.

We are promoting walking as a means of increasing PA, and the duration of the trial for participants is 3 months. Evidence from our previous trial suggested that the risk of harm or injury to a patient associated with our trial is low. We do not foresee any events or circumstances in which we would need to stop the trial prematurely. No interim analysis or stopping guidelines have been made prior to the study. However, a trial steering committee made up of an independent chair, three independent members, including members of the Public and Patient Involvement (PPI) panel, representatives from the funder and the sponsor, and three members of the research team will oversee all aspects of the trial.

Data analysis will be undertaken on completion of data collection, database lock and data cleaning.

#### Confidentiality

The research team will have no access to identifiable data prior to consent. Study invitations are sent directly from GP practices. The storage and movement of identifiable and sensitive data between GP practices and the research team will be undertaken following the guidelines and principles of Good Clinical Practice, the Data Protection Act 1998 and the NHS England confidentiality policy. The chief investigator will be the custodian of the trial data set, and only authorised personnel will have access to the (anonymous) data.

### Study outcomes

Data are collected by nurses and healthcare assistants during the NHS Health Check and from questionnaires and accelerometer wear at 3 months post-NHS Health Check. See Table 2 for a full list of outcome measures.

**Table 2:** Study measures

	0 months	3 months
Accelerometer measures		
Activity counts per minute		X
Step counts per day		X
Time (minutes/day) in light/sedentary activity		X
Time (minutes/day) in moderate activity		X
Time (minutes/day) in vigorous activity		X
Time (minutes/day) in moderate or vigorous activity		X
Questionnaire measures		
Physical activity energy expenditure (PAEE) (kj/kg/day)		X
Home-based PAEE (kj/kg/day)		X
Work-based PAEE (kj/kg/day)		X
Leisure-based PAEE (kj/kg/day)		X
Commuting PAEE (kj/kg/day)		X
Screen/TV time (hours per day)		X
Gender	X	
Age (calculated from date of birth)	X	
Ethnicity	X	
Education	X	
Employment status	X	
Household income	X	
Marital status	X	
Home ownership	X	
Vehicle ownership	X	
Dependents	X	
Individual deprivation score	X	
Area deprivation score (Index of Multiple Deprivation, IMD, based on home postcode)	X	
Recall of physical activity (PA) advice		X
PA awareness		X
Use of intervention materials		X
Enactment of behaviour change techniques		X
Contamination of sample		X
Primary care visits		X
Hospital visits		X
Out of pocket expenditure		X
Work productivity		X
National Health Service (NHS) Health Checks		
10-year cardiovascular risk score (QRisk2)	X	
Activity level (Derived from GPPAQ)	X	
Duration of health check	X	
Nurse/Healthcare assistant delivery	X	
Physical activity referrals	X	

#### Primary outcome

The primary outcome is PA (total body movement) measured by tri-axial accelerometry (Actigraph GT3X+ or Actigraph w-GT3X-BT, ActiGraph, Pensacola, Florida, USA) expressed as average vector magnitude acceleration (counts per minute). Data collected at 60Hz will be integrated into 10-second epochs. Non-wear time, defined as strings of 90 minutes of consecutive zeros (on the vertical axis), will be excluded, and the remaining vector magnitude data will be summarised into average acceleration (counts per minute (cpm)).

#### Secondary outcomes

Secondary outcomes derived from the accelerometer data are step counts (average step counts per day) and the average number of minutes per day spent in sedentary/light activity (<2,690 cpm); moderate activity (2,690–6,166 cpm); vigorous activity (≥6,167 cpm); and moderate or vigorous activity (≥2,690 cpm) [22].

Self-reported PA outcome measures will be obtained using the validated Recent PA Questionnaire (RPAQ) [20]. Total Physical Activity Energy Expenditure (PAEE), domain-specific PAEE (home, work, leisure-time and commuting) and screen/TV viewing time over the past 4 weeks will be calculated using reported frequency and duration for each activity, together with estimated activity-specific metabolic cost [23].

The cost analysis will inform an economic evaluation and will comprise costs to participants, the NHS (including the cost of the intervention itself), and lost productivity. Costs to participants will be calculated from out-of-pocket expenditure reported in questionnaires, adjusted to the price year of the analysis using the consumer price index (CPI). The value of lost productivity will be calculated from the WPAI [21]. This estimates reduced productivity whilst at work (‘presenteeism’) as a self-assessed proportion of maximum productivity, as well as days absent from work. The cost will be calculated as the sum of whole-day equivalents of work lost multiplied by the median daily wage in England rate for the price year of the analysis. The cost to the NHS will be calculated as the sum of self-reported primary and secondary care contacts multiplied by representative unit costs pertaining to the price year of the analysis (e.g. NHS Reference Costs [24] and Curtis [25]). Finally, the cost to the NHS of providing the intervention will be calculated from study records of expenditure on pedometers, materials and training and the time required to deliver the VBI.

#### Process measures

The process evaluation is informed by recent Medical Research Council guidance [26] and will focus on the mechanisms underlying any intervention effects and the implementation (fidelity of delivery) of intervention and control consultations.

##### Mechanisms of impact

Variables hypothesised to be on the causal pathway of any effects include (1) recall of any PA advice received during the NHS Health Check consultation (two items); (2) awareness of PA recommendations and own PA levels (two items); (3) the use of any materials handed out (e.g. booklet; three items); and (4) enactment of key behaviour change techniques included in the intervention (e.g. goal setting (behaviour) and self-monitoring (behaviour); four items).

##### Fidelity of delivery

A reliable coding frame will be developed and piloted for inter-rater reliability to assess the following variables: (1) duration of the NHS Health Check and the intervention (if applicable); (2) delivery of the standard NHS Health Check (e.g. taking a blood sample or measuring blood pressure); (3) delivery of the intervention (e.g. feedback on PA levels, mention PA recommendations, explain the pedometer, prompt goal setting and self-monitoring, and give a booklet); and (4) contamination, by coding of any intervention-related components in the control consultations.

### Sample size

A trial of 394 participants per arm followed up is sufficient to detect a 0.2 sd (‘small’) difference in mean activity between arms (40 accelerometer cpm) based on the standard deviation of 200 cpm estimated in the previous trial) with 80 % power. However, allowing for attrition of 30 % at follow-up (i.e. 30 % of participants not providing sufficient accelerometer data), an initial sample size of 570 per arm would give 80 % power to detect an effect of this size between the two conditions (alpha = 0.05, two-sided test).

Recruitment and attrition will be monitored. If attrition is lower than 30 % and we anticipate achieving follow-up of 394 participants per arm with sufficient accelerometer data from randomising fewer than 570 per arm, participant recruitment will be closed.

### Statistical analyses

Full details of the statistical methods are provided in the statistical analysis plan. The following is a summary of the main analyses proposed.

We will use analysis of covariance to test for intervention effects on continuous outcomes and quantify these with differences in means and 95 % confidence intervals, adjusting for primary care practice, gender and age. Logistic regression will be used for binary outcomes. An intention-to-treat approach will be used, supported by a per protocol analysis for the primary outcome analysis. All significance tests will be two-sided and assessed at the 5 % level of significance.

Missing data in the primary outcome will be handled within a sensitivity analysis considering optimistic and pessimistic scenarios for the intervention effect size in those with missing data and incorporating baseline predictors of primary outcome missing status that are differential by group. This analysis will be undertaken to examine the robustness of the main analysis result to the ‘missing at random’ (MAR) assumption. The aim is to adequately explore the impact of departures from the MAR assumption on the primary outcome results [27].

Pre-specified subgroup variables will be examined in relation to the primary outcome and will involve an initial test of differential intervention effect across the subgroup variable before summarising the intervention effect within the subgroup categories. These subgroup variables will include baseline cardiovascular risk, gender, age (40–59; 60–74 years), ethnic group, educational qualifications, employment status, household income, marital status, home ownership, vehicle ownership, and deprivation score (Index of Multiple Deprivation (IMD) 2007, derived from the participant’s home postcode [28]). For a continuous moderator such as CVD risk, the intervention effect observed in the highest tertile of the moderator will be estimated with a 95 % confidence interval having an informative width of +/- 25 thousand activity counts per day.

Incremental effectiveness will also be formally integrated with incremental cost data forming a within-trial economic evaluation. This will estimate the incremental cost per incremental MET(Metabolic Equivalent of Task)-hour of physical activity gained (estimated from accelerometer counts). The analysis will be reported from the perspectives of the NHS and society. Current guidelines on the conduct and reporting of economic evaluations will be followed [29–31]. Analysis of uncertainty will comprise the estimation of 95 % confidence intervals around incremental costs and outcomes and construction of the cost-effectiveness acceptability curves.

In addition to the within-trial analysis, results will be combined with prior data on the costs and effects of the intervention, and a previously developed model will be updated with the new evidence to predict the longer-term costs and outcomes associated with the intervention and control. This will yield a revised estimate of the incremental cost per QALY gained. The revised decision uncertainty will be reported as an updated cost-effectiveness acceptability curve.

Questionnaire measures relating to the proposed mechanisms of impact of the intervention (recall of PA advice, awareness of PA, use of intervention materials, and enactment of behaviour change techniques) will be analysed descriptively. Variables that assess fidelity of the intervention and control consultations, derived from coding the audio-recordings, will also be analysed descriptively. Two overall fidelity scores will be calculated, expressed as the number of components delivered out of those that should be delivered for (1) the NHS Health Check (intervention and control consultations) and (2) the intervention (intervention consultations only).

## Discussion

The main aims of the VBI trial are to estimate the effectiveness and the cost-effectiveness of the Step It Up intervention delivered at the end of an NHS Health Check compared with the Health Check alone. The findings of this trial will extend the evidence base on very brief interventions for PA and will inform policy decisions about the introduction of very brief PA interventions into primary care consultations.

The trial has been designed to maximise both internal and external validity. The participant is randomised by a web-based program during the consultation. At point of entry into the trial (i.e. as informed consent is obtained), neither the participant nor the practitioner is aware of the group allocation. Once the participant is allocated, the practitioner tells them which group they are in. In a trial such as this, practitioners and participants cannot be blinded to the group allocation.

With individual randomisation of participants (as opposed to cluster randomisation of practitioners or practices), the risk that some of the content of the very brief intervention may be incorporated into the Health Check and that participants in the control arm may therefore receive some intervention content does exist. However, this did not occur in our trial of a practice nurse-delivered intervention for medication adherence [32]. The CRF and the written Step It Up intervention material have been designed to act as a prompt and guide to the consultation, thus minimising the risk of contamination. Audio-recording a sample of consultations will enable fidelity of delivery to be checked.

The primary outcome measure for this trial is PA objectively measured using an accelerometer at 3-month follow-up and expressed in activity counts per minute. Analysis of accelerometer data will be blinded. Secondary outcomes include self-report measures of PA, which provide information on the type and pattern of activity not otherwise obtainable from accelerometry.

After careful consideration, we decided not to measure the primary outcome at baseline. A baseline measure would have a number of advantages: sample size could be reduced, and an analysis of individual change scores would be possible. However, pilot work undertaken in preparation for this trial identified two potential problems arising from objective measurement of PA at baseline. First, baseline measurement is logistically difficult and may compromise uptake of the NHS Health Check and the flow of participants through the trial. Second, baseline accelerometry could act as an intervention; that is, some participants may increase their activity as a result of wearing the device, leaving less scope for our very brief intervention to have an effect [33].

The Step It Up intervention was chosen after a rigorous selection process [10] and evaluation in a previous trial (Pears S, Bijker M, Morton K, Vasconcelos J, Parker RA, Westgate K, et al.: A randomised controlled trial of three very brief interventions for physical activity in primary care, submitted) (ISRCTN02863077). The intervention is well characterised in terms of the component BCTs, is feasible to deliver in practice with limited training and is acceptable to both practitioners and participants. External validity is further increased by using nurses and healthcare assistants already trained to carry out NHS Health Checks.

The trial team brings together expertise in behavioural science, intervention development and evaluation, measurement of PA, medical statistics, health economics, and the design, conduct and analysis of primary care trials. In designing this trial, we have drawn on our previous work and on the guidelines and principles of trial reporting laid out in SPIRIT [34] and CONSORT [35]. See Additional file 1 for the SPIRIT Checklist.

## Trial status

Participant recruitment for this trial is underway.

## Abbreviations

BCT, behaviour change technique; CMO, chief medical officer; CRF, case report form; GP, general practice or general practitioner; GPPAQ, GP PA questionnaire; ICER, incremental cost-effectiveness ratio; IMD, Index of Multiple Deprivation; MAR, missing at random; MET, Metabolic Equivalent of Task; NHS, National Health Service; NICE, National Institute for Health and Care Excellence; PA, physical activity; PAEE, physical activity energy expenditure; PPI, patient and public involvement; RPAQ, Recent Physical Activity Questionnaire; VBI, very brief intervention; WPAI, Work Productivity and Activity Impairment

## Additional file

Additional file 1: SPIRIT Checklist. (PDF 130 kb)

## Footnotes

1 Two models of Actigraph accelerometers (GT3X+ and w-GT3X+) were used in this trial. Unfortunately, during the previous trial a number of accelerometers were ‘lost’ (either in transit or were never returned), and as a result more accelerometers were purchased to make up the deficit. By this time, a new generation of Actigraph accelerometers had been produced. The manufactures claim the data provided by the GT3X+ and w-GT3X+, when subjected to the same acceleration environment, will exhibit outputs within 5 % of each other, and with randomisation, these are expected to be represented in the same proportions in the two randomised groups.
